# Impact of age on the host response to sepsis in a murine model of fecal-induced peritonitis

**DOI:** 10.1186/s40635-024-00609-8

**Published:** 2024-03-08

**Authors:** Neha Sharma, Alex Chen, Leah Heinen, Ruth Liu, Dhruva J. Dwivedi, Ji Zhou, Manoj M. Lalu, Asher A. Mendelson, Braedon McDonald, Colin A. Kretz, Alison E. Fox-Robichaud, Patricia C. Liaw

**Affiliations:** 1 Thrombosis and Atherosclerosis Research Institute (TaARI), 237 Barton St E., Room C5-107, Hamilton, ON L8L 2X2 Canada; 2 McMaster University, Hamilton, ON Canada; 3 Department of Medical Sciences, McMaster University, Hamilton, ON Canada; 4 Department of Medicine, McMaster University, Hamilton, ON Canada; 5 Regenerative Medicine Program, Ottawa Hospital Research Institute, Ottawa, ON Canada; 6 Clinical Epidemiology Program, Blueprint Translational Group, Ottawa Hospital Research Institute, Ottawa, ON Canada; 7 Department of Anesthesiology and Pain Medicine, Department of Cellular and Molecular Medicine, The Ottawa Hospital, Ottawa, ON Canada; 8 Section of Critical Care Medicine, Department of Medicine, Rady Faculty of Health Sciences, University of Manitoba, Winnipeg, MB Canada; 9 Snyder Institute for Chronic Diseases, Cumming School of Medicine, University of Calgary, Calgary, AB Canada; 10 Department of Critical Care Medicine, Cumming School of Medicine, University of Calgary, Calgary, AB Canada

**Keywords:** Sepsis, Age, Fecal-induced peritonitis model, Immunothrombosis, National Preclinical Sepsis Platform

## Abstract

**Introduction:**

Despite older adults being more vulnerable to sepsis, most preclinical research on sepsis has been conducted using young animals. This results in decreased scientific validity since age is an independent predictor of poor outcome. In this study, we explored the impact of aging on the host response to sepsis using the fecal-induced peritonitis (FIP) model developed by the National Preclinical Sepsis Platform (NPSP).

**Methods:**

C57BL/6 mice (3 or 12 months old) were injected intraperitoneally with rat fecal slurry (0.75 mg/g) or a control vehicle. To investigate the early stage of sepsis, mice were culled at 4 h, 8 h, or 12 h to investigate disease severity, immunothrombosis biomarkers, and organ injury. Mice received buprenorphine at 4 h post-FIP. A separate cohort of FIP mice were studied for 72 h (with buprenorphine given at 4 h, 12 h, and then every 12 h post-FIP and antibiotics/fluids starting at 12 h post-FIP). Organs were harvested, plasma levels of Interleukin (IL)-6, IL-10, monocyte chemoattract protein (MCP-1)/CCL2, thrombin-antithrombin (TAT) complexes, cell-free DNA (CFDNA), and ADAMTS13 activity were quantified, and bacterial loads were measured.

**Results:**

In the 12 h time course study, aged FIP mice demonstrated increased inflammation and injury to the lungs compared to young FIP mice. In the 72 h study, aged FIP mice exhibited a higher mortality rate (89%) compared to young FIP mice (42%) (p < 0.001). Aged FIP non-survivors also exhibited a trend towards elevated IL-6, TAT, CFDNA, CCL2, and decreased IL-10, and impaired bacterial clearance compared to young FIP non-survivors.

**Conclusion:**

To our knowledge, this is the first study to investigate the impact of age on survival using the FIP model of sepsis. Our model includes clinically-relevant supportive therapies and inclusion of both sexes. The higher mortality rate in aged mice may reflect increased inflammation and worsened organ injury in the early stage of sepsis. We also observed trends in impaired bacterial clearance, increase in IL-6, TAT, CFDNA, CCL2, and decreased IL-10 and ADAMTS13 activity in aged septic non-survivors compared to young septic non-survivors. Our aging model may help to increase the scientific validity of preclinical research and may be useful for identifying mechanisms of age-related susceptibility to sepsis as well as age-specific treatment strategies.

**Supplementary Information:**

The online version contains supplementary material available at 10.1186/s40635-024-00609-8.

## Introduction

Sepsis is the leading cause of death in non-coronary intensive care unit patients and is recognized as a global health priority by the World Health Organization [1–3]. Although sepsis can affect individuals of all ages, case-fatality rates increase linearly by age [4]. Compared with young adults, the case-fatality rates for middle-aged and elderly adults are approximately twofold and 2.5-fold higher, respectively [4]. Older patients also die earlier during hospitalization and older survivors often experience long-term cognitive impairment and functional disability [4, 5].

The increased risk of sepsis associated with aging is likely multifactorial and may include factors such as immunosenescence, deficiencies in both innate and adaptive immunity, altered cytokine production, abnormal coagulation, and increased prevalence of comorbid diseases [6–8]. Immunosenescence is the inability of the aged immune system to mount an effective response to pathogens as compared to the young, placing older adults at a higher risk of developing an infection [9, 10]. Altered functions of the innate immune system include age-related alteration of neutrophils and macrophages such as reduced chemotaxis, phagocytosis, antibacterial defense, and formation of reactive oxygen species [6, 11]. Altered functions of the adaptive immune system include a reduction in B cell number and generation of naïve T cells, resulting in a reduced ability to respond to new pathogens [6, 12]. Furthermore, an abnormal cytokine response in the elderly shifts formation of type 1 cytokines to type 2 cytokines, predisposing the elderly to systemic infection by pathogens and prolonged proinflammatory responses as compared to younger patients [9, 13]. Aging can also lead to an increase in plasma levels of fibrinogen, factor VII, factor VIII, factor IX, and plasminogen activator inhibitor 1, which are further augmented during sepsis and contribute to the increased risk of thrombosis in the elderly [9, 10].

Due to the high cost of maintaining colonies of older animals, preclinical sepsis studies commonly use mice that are less than 3 months old, which is comparable to a 20-year-old person [6]. This results in decreased scientific validity since age is an independent predictor of mortality in sepsis [6, 9]. In fact, less than 1% of pre-clinical sepsis studies utilize appropriately aged animals [6]. To study sepsis in aged mice, most investigators utilize the lipopolysaccharide (LPS) injection model or the cecal ligation and puncture (CLP) model [6]. In addition to differences in model systems, there is also a lack of standardization or inclusion of treatments that mimic the clinical treatment of sepsis (ie. fluid resuscitation and antibiotics) [6, 14, 15]. Furthermore, most preclinical mouse studies utilize male animals, which limits the generalizability of the studies [14, 15].

To improve the quality of preclinical sepsis research, we established the National Preclinical Sepsis Platform (NPSP), one of the research pillars supported by Sepsis Canada that is funded by the Canadian Institutes of Health Research (CIHR) [15]. As members of the NPSP, we created a multicentre infrastructure to rigorously study the pathophysiology of sepsis and to accelerate the movement of promising therapies into early phase clinical trials [15]. We have established and optimized a 72 h model of abdominal sepsis using fecal-induced peritonitis (FIP) [16].

The purpose of this study is to investigate the impact of aging on the host response to sepsis using the standardized FIP model that has been developed by the NPSP. The current study utilizes 3-month-old young mice (~ 20 years in humans) and 12-month-old middle-aged mice (~ 50 years in humans) [6, 17]. To avoid the confounding effect of obesity with age, we used a diet restriction model of aging as previously described with minor modifications [18]. In addition to assessing sepsis survival and organ histology, we quantified age-related differences in immunothrombosis, a process in which pathogen-induced immune activation leads to increased inflammation, blood clotting, and the formation of neutrophil extracellular traps (NETs) [19]. Uncontrolled immunothrombosis is one of the hallmarks of sepsis that can lead to tissue hypoperfusion, organ failure, and death [19–21]. We hypothesize that aged mice will exhibit a lower survival rate and increased organ injury in response to sepsis compared to young mice.

## Methods

### Animals

All mice received humane care in accordance with Canadian Council on Animal Care (CCAC) guidelines. This study was approved by the McMaster Animal Research Ethics Board. This study is reported in accordance with the ARRIVE 2.0 guidelines (Additional file 2: Appendix S1). Male and female C57BL/6 mice (Helicobacter hepaticus-free) were purchased from Charles River Laboratories (Sherbrooke, Quebec, Ontario, Canada) and placed in standard housing in the Animal Care Facility at the Thrombosis and Atherosclerosis Research Institute (TaARI) at McMaster University (Hamilton, Ontario, Canada). Mice were housed in a Helicobacter- and Norovirus-negative clean room in HEPA-filtered ventilated cages (Tecniplast Sealsafe Plus system) under 12 h dark/light cycles. Cages contained corncob bedding, nesting material, a structure (e.g. plastic igloos), and autoclaved bottled water (provided via the Avidity Life Sciences Reverse Osmosis 8600 system).

### Diet restriction model of aging

Starting at approximately 8 months of age, the mice were placed on diet restriction according to the aging protocol described by Gill et al., with minor modifications [18]. Briefly, food pellets (Teklad Irradiated Global 18% Protein Rodent Diet 2918) were provided to the mice ad libitum and the amount of food consumed was measured over 2 weeks. The amount of food provided to the mice was then reduced by 10% (of the measured amount), which was maintained for the duration of the aging process (to 12 months of age).

#### Fecal-induced peritonitis (FIP) model of abdominal sepsis

The rat fecal slurry was prepared according to the FIP protocol described by Sharma et al. [16]. The rat fecal slurry was injected into healthy male and female C57BL/6 mice, which were either 3 months or 12 months old. The required aliquots of fecal slurry were thawed and warmed to room temperature prior to injection. The mice received intraperitoneal (IP) injections of 0.75 mg/g of fecal slurry according to body weight. Control mice received IP injections of 5% dextrose (with 10% glycerol). The average weights of the young mice and the diet-restricted aged mice were 24.6 ± 3.1 g and 28.3 ± 2.3 g, respectively. Following injection, mice from the same treatment groups were kept together and returned to their cages with bedding, enrichment, autoclaved water, and allowed to recover. External heat was provided for all mice through heating blankets placed below half of each cage to allow mice to regulate their own body temperature [16, 22].

#### Modified murine sepsis score (MSS)

 Modified murine sepsis score (MSS) was used to assess sepsis severity [16, 22]. The modified MSS involves observing posture, respiration quality, responsiveness, activity, and appearance [16, 22]. The MSS component scores were standardized to a four-point scale ranging from 0 (healthy) to 3 (sick) (Additional file 1: Table S1). Mice were humanely euthanized if their average MSS was ≥ 1.75, if any component of MSS was equal to 3, or if they reached the end of the study.

### Temperature monitoring

We measured body temperature using contactless temperature chips that were subcutaneously inserted beneath the dorsal skin in the neck (United Information Devices, Lake Villa, Illinois, USA).

#### 12 h and 72 h FIP studies

To investigate time-dependent changes in disease severity during the early phase of sepsis, young and aged FIP and sham mice were monitored and culled at the following timepoints: 4 h, 8 h, and 12 h (outlined in Additional file 1: Table S2). In a separate cohort of mice (FIP, sham, and naive), the animals were monitored for 72 h and were euthanized at humane endpoint or at study endpoint (outlined in Additional file 1: Table S2). Mice in all cohorts were randomly selected from their cages and randomly allocated to the experimental groups. Fluids, antibiotics, and analgesia were administered to the mice, consistent with standard practice for the treatment of human sepsis and current recommendations [14, 16]. For both studies, the mice were injected with 0.05 mg/kg of buprenorphine (subcutaneous) at 4 h. For the 72 h study, the mice received antibiotics (25 mg/kg of imipenem IP), Ringer’s lactate (15 mL/kg subcutaneous), and 0.05 mg/kg of buprenorphine at 12 h and every 12 h until study endpoint.

#### Collection of blood and peritoneal cavity fluid

At humane or experimental endpoint, mice were anesthetized with isoflurane and oxygen inhalation. Phosphate buffered saline (PBS) was injected into the peritoneal cavity and peritoneal cavity fluid (PCF) was collected. Blood was collected via the inferior vena cava into a one tenth volume of 3.2% buffered citrate. Plasma was prepared by centrifugation at 5000 × *g* for 10 min (twice), aliquoted, and stored at − 80 °C.

#### Quantification of bacterial cultures 

At humane or experimental endpoint, bacterial loads were assessed in the PCF and blood. Ten-fold serial dilutions of PCF and blood in phosphate buffered saline were prepared until 10,000X dilution. Ten μL of each dilution starting from 10X were spotted in triplicate on 5% blood agar plates. Agar plates were placed at 37 °C overnight and colonies from the highest dilution were counted.

### Quantification of lung myeloperoxidase, interleukin-6, interleukin-10, thrombin-antithrombin complexes, cell-free DNA, monocyte chemoattract protein (MCP-1)/CCL2, and ADAMTS13 activity

Levels of lung MPO were quantified using a mouse MPO DuoSet (R&D Systems, Minneapolis, Minnesota, USA). Plasma levels of Interleukin (IL)-6, IL-10, and monocyte chemoattract protein (MCP-1)/CCL2 were measured using the mouse IL-6 Duoset, the mouse IL-10 quantikine ELISA, and the Mouse CCL2/JE/MCP-1 DuoSet respectively, (R&D Systems, Minneapolis, Minnesota, USA). Plasma levels of thrombin antithrombin (TAT) complexes were quantified using the matched-pair antibody set (Affinity Biologicals, Ancaster, Ontario, Canada) according to the manufacturer’s protocol. CFDNA was quantified as per the manufacturer’s instructions using their Quant-iT™ PicoGreen™ assay (ThermoFisher Scientific, Waltham, Massachusetts, USA). ADAMTS13 activity was measured using FRETS-VWF73 (Anaspec Inc, Fremont, California, USA).

#### Organ histology 

Histology was performed on the lung, liver, and kidneys. Organ sections were stained with hematoxylin and eosin (H&E). The sections were scored in a blinded fashion by two individuals, on a scale of 0 to 5 (0 = normal, 5 = severe) based on inflammation, thrombosis, and organ morphology. A composite score (organ damage) was calculated as a sum of the 3 categories (described in Additional file 1: Table S3).

#### Statistical analysis

Statistical analyses were performed using GraphPad Prism version 9.1.1 for macOS (GraphPad Software, San Diego, California, USA, www.graphpad.com). Values were expressed as mean ± standard deviation (SD) and p-values < 0.05 were considered significant. Significant differences between groups were determined using an ordinary one-way analysis of variance (ANOVA) or mixed-effects analysis. For the 72 h study, since there was only one mouse that survived in the aged FIP group, the aged FIP survivor group (n = 1) was not included in the statistical analysis. Survival curves were analyzed using a Log-rank (Mantel-Cox) test. 

## Results

### Time-dependent changes in Murine Sepsis Score (MSS), body temperature, and bacterial loads during the early stage of sepsis

**Fig. 1:**
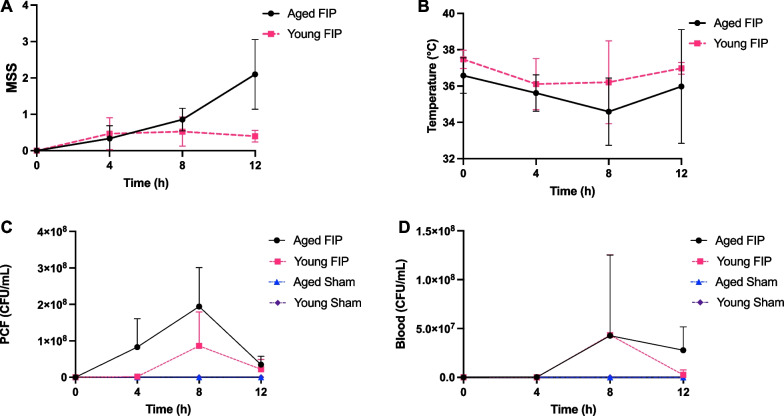
Time-dependent changes in MSS, body temperature, and bacterial loads in aged and young mice during the early phase of sepsis (12 h time course study). MSS (**A**), temperature (**B**), bacterial loads in PCF (**C**), and bacterial loads in blood (**D**) over time in aged and young FIP mice. Bacterial loads: aged FIP (n = 4–6/timepoint), young FIP (n = 4–6/timepoint), aged sham (n = 3/timepoint), and young sham (n = 3/timepoint). (Note: Data are presented as mean ± SD. (Note: For 1 aged FIP mouse, blood was not collected. For 1 young FIP mouse PCF and blood colonies were too confluent to count). Data are presented as mean ± SD

In this study, sepsis was induced in young and aged mice via IP injections of 0.75 mg/g of rat fecal slurry. Control mice received IP injections of control vehicle. To study the early stage of sepsis, the mice were culled at 4 h, 8 h, or 12 h. During this 12 h time frame, the animals received buprenorphine at 4 h. As shown in Fig. 1A, aged FIP mice demonstrated a robust increase in the MSS (described in Additional file 1: Table S1) at 12 h post-FIP compared to young FIP mice (Fig. 1A). Both aged and young FIP mice experienced a decrease in body temperature at 4 h post-FIP, with a trend towards lower body temperatures in the aged FIP mice (Fig. 1B).

To quantify bacterial loads in the blood and peritoneal cavity, blood and PCF were collected at 4 h, 8 h, or 12 h post-FIP/control vehicle injection. Compared to young FIP mice, aged FIP mice exhibited a trend towards higher bacterial loads in the PCF at 4 h and 8 h and in the blood at 12 h (Fig. 1C, D). As expected, there was no/minimal bacterial growth in the PCF and blood of age-matched sham mice (Fig. 1C, D). This data suggests that there is an age-related reduction in bacterial clearance during the early phase sepsis.

### Time-dependent changes in immunothrombosis biomarkers, lung MPO, chemoattractant mediator, and organ injury during the early stage of sepsis

**Fig. 2:**
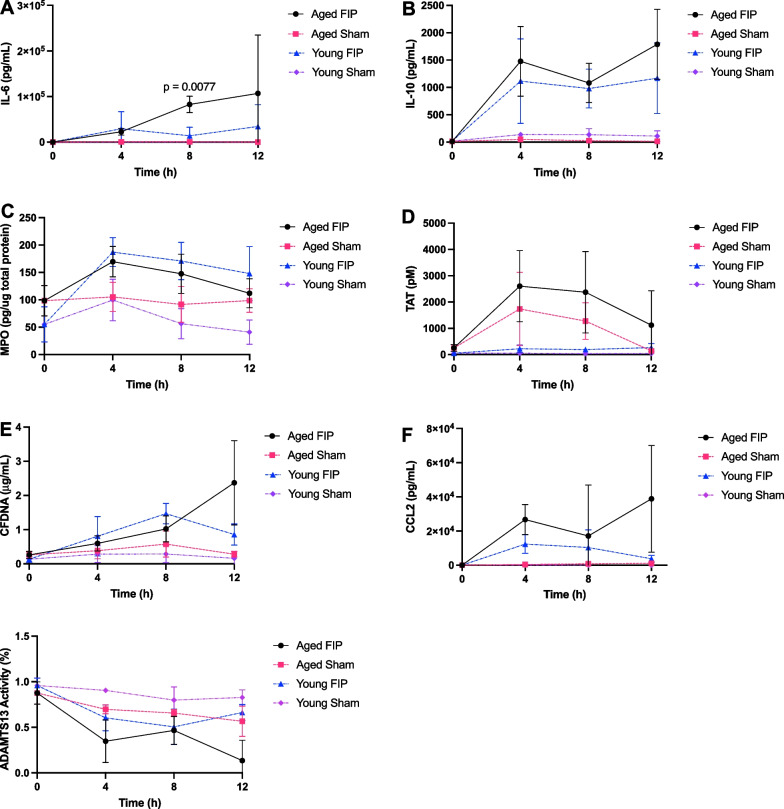
Longitudinal changes in IL-6, IL-10, lung MPO, TAT, CFDNA, CCL2, and ADAMTS13 activity in aged and young mice during the early phase of sepsis (12 h time course study). IL-6 (**A**), IL-10 (**B**), lung MPO (**C**), TAT (**D**), CFDNA (**E**), CCL2 (**F**), and ADAMTS13 activity (**G**) over time in aged and young FIP and sham mice. Aged FIP (n = 4–6/timepoint), young FIP (n = 4–6/timepoint), aged sham (n = 3–6/timepoint), and young sham (n = 3–6/timepoint). (Note: Data are presented as mean ± SD. The data was analyzed using a mixed-effects analysis. P-values < 0.05 were considered significant

Next, we investigated time-dependent changes in plasma levels of immunothrombosis biomarkers, neutrophil infiltration in the lungs (as assessed by lung MPO), as well as the chemoattractant mediator CCL2 during the early phase of sepsis. As expected, IL-6, IL-10, lung MPO, TAT, CFDNA, and CCL2 in the sham mice were all low (Fig. 2A–F) and ADAMTS13 activity was high (Fig. 2G) compared to their age-matched FIP mice. At 8 h post-FIP, aged FIP mice exhibited elevated levels of IL-6 compared to young FIP mice (Fig. 2A). There were no differences in IL-10, lung MPO, TAT, CFDNA, CCL2, and ADAMTS13 activity between aged FIP mice and young FIP mice in the early stage of sepsis (Fig. 2B–G).

**Fig. 3:**
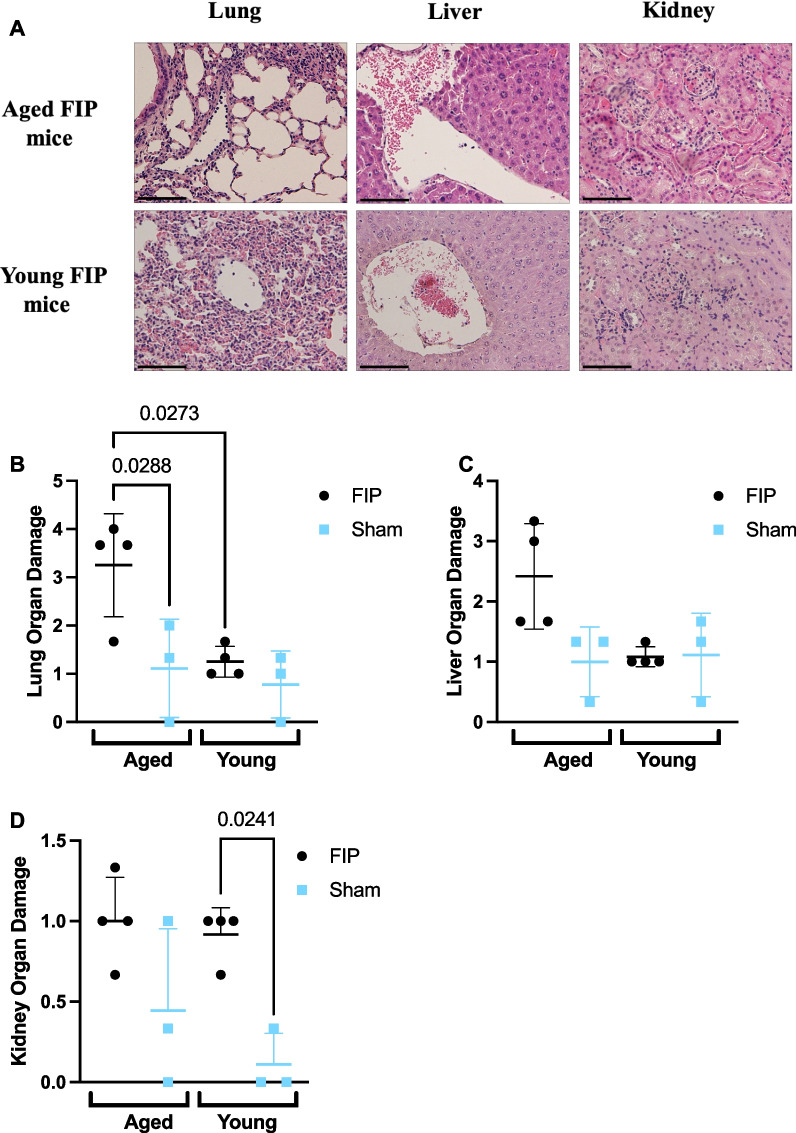
Histology scores in the lung, liver, and kidney in aged and young mice during the early phase of sepsis (12 h time course study). Representative images of the lung, liver, and kidney sections stained for H&E in aged and young mice at 8 h post-FIP (**A**). Organ injury scores for the lung (**B**), liver (**C**), and kidney sections (**D)** were based on inflammation, thrombosis, and organ morphology. Aged FIP (n = 4), young FIP (n = 4), aged sham (n = 3), and young sham (n = 3). Data are presented as mean ± SD and analyzed using a one-way ANOVA. P-values < 0.05 were considered significant. Scale bars represent 50 $\mu$ m

To assess organ injury, the lung, liver, and kidney were stained with H&E and scored based on organ morphology, inflammation, and thrombosis. At 8 h post-slurry injection, aged FIP mice exhibited elevated levels of lung injury compared to young FIP mice (Fig. 3A, B). There was no difference in liver and kidney injury between aged FIP mice and young FIP mice (Fig. 3A–D). Taken together, these results suggest that aged FIP mice experience increased inflammation (IL-6) as well as increased injury to the lungs during the early stage of sepsis compared to young FIP mice.

### Survival, physiological parameters, and bacterial loads in a 72 h FIP model of sepsis

**Fig. 4:**
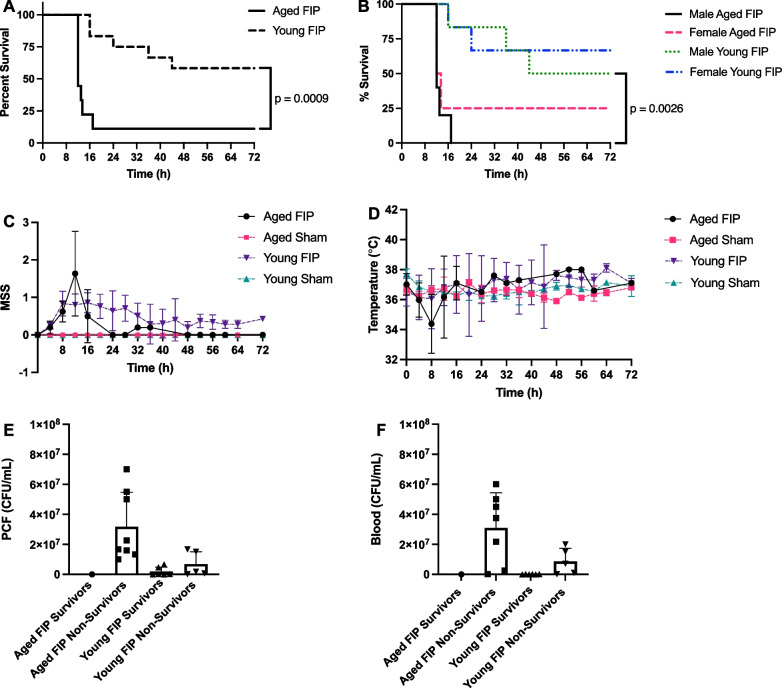
Effects of age on mortality, MSS, and body temperature in a 72 h FIP model of sepsis. Kaplan–Meier survival curves (**A** and **B**), MSS (**C**), and body temperature (**D**) over time in aged and young FIP mice. Mice that survived till experimental endpoint are designated as survivors. Mice that were humanely euthanized according to their MSS are designated as non-survivors. Aged FIP (n = 9), young FIP (n = 12), aged sham (n = 3), and young sham (n = 3). Data are presented as mean ± SD. Survival curves were analyzed using a Log-rank (Mantel-Cox) test. Bacterial loads were analyzed using a one-way ANOVA. P-values < 0.05 were considered significant. Bacterial loads for PCF (E) and blood (F) in aged and young FIP survivors and non-survivors. (Note: For 1 aged FIP non-survivor, unable to collect blood and for 1 young FIP survivor, blood and PCF colonies were too confluent to count).

To determine the effect of age on infection-induced mortality, another cohort of young (n = 12) and aged mice (n = 9) were subjected to FIP (using the same slurry dose of 0.75 mg/g) and survival was monitored for 72 h. Mortality resulted from a combination of natural disease progression and humane euthanasia based on our surrogate makers of death. As shown in Fig. 4A, the mortality rate in the aged FIP mice and young FIP mice was 89% and 42%, respectively (p < 0.001), with aged mice dying earlier. When stratified by sex, the mortality rate in the aged male mice and young male mice was 100% and 50%, respectively (Fig. 4B, p = 0.003). Similarly, aged female mice had a higher mortality rate compared with young female mice (75 vs 33%, Fig. 4B), but the difference was not statistically significant (p = 0.08). Although male mice had higher mortality rates compared to female mice in both young and aged cohorts, this difference was not statistically significant. Overall, based on these survival studies, aged mice demonstrated a higher mortality rate compared to young mice.

During the 72 h time frame, all mice were monitored every 4 h (according to Additional file 1: Table S1), until reaching humane or study endpoint. As shown in Fig. 4C, aged FIP mice exhibited an increase in MSS that peaked at 12 h, after which time the MSS returned to low values. As shown in Fig. 4D, the body temperature in all mice dropped within the first 8 h post-slurry injection, with aged mice experiencing a greater drop in body temperature compared to young mice at 8 h. These results suggest that within the first 8 to 12 h of sepsis, aged FIP mice exhibit increased disease severity and lower body temperatures compared to young FIP mice. Sex-based differences in MSS and temperature are shown in Additional file 1: Fig. S1.

Next, we determined if age impacts the levels of bacterial load in the PCF and blood in our 72 h model of sepsis. The mice were stratified into survivors and non-survivors. As shown in Figs. 4E, F, aged FIP non-survivors had the highest bacterial loads followed by young FIP non-survivors. As expected, both young and aged survivors had virtually no bacterial growth in their blood and PCF (Fig. 4E, F). This data suggests that aged FIP mice experience reduced bacterial clearance during sepsis compared with young FIP mice. Sex-based differences in bacterial loads are shown in Additional file 1: Fig. S1.

### Immunothrombosis biomarkers, lung MPO, chemoattractant mediator, and organ injury in a 72 h FIP model of sepsis

To examine the effects of age on immunothrombosis biomarkers in our 72 h FIP model of sepsis, blood was collected from aged and young septic mice (in survivors as well as non-survivors). It should be noted that only one out of nine aged mice survived. Thus, the data from the single aged survivor mouse should be interpreted as preliminary data. To account for the effects of aging, blood was also collected from age-matched sham and naïve mice.

**Fig. 5:**
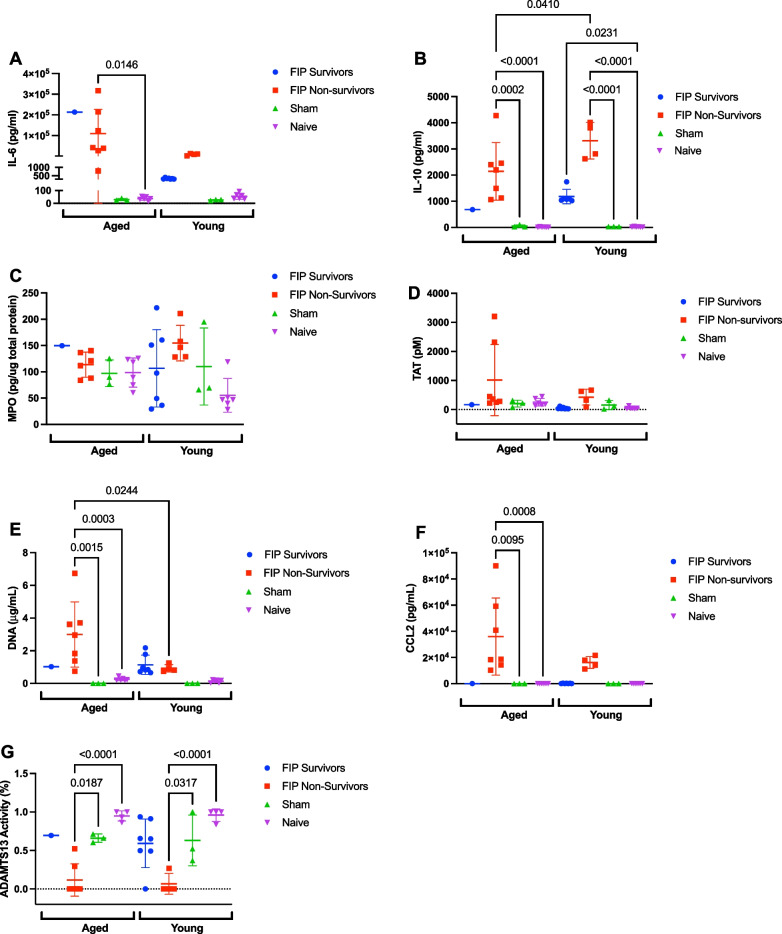
Effects of age on plasma levels of IL-6, IL-10, TAT, CFDNA, CCL2, ADAMTS13 activity, and lung MPO in a 72 h FIP model of sepsis. IL-6 (A), IL-10 (B), MPO (C), TAT (D), CFDNA (E), CCL2 (F), and ADAMTS13 activity (G) over time in aged and young FIP survivors and non-survivors and in sham and naive mice. Mice that survived till experimental endpoint are designated as survivors. Mice that were humanely euthanized according to their MSS are designated as non-survivors. Aged FIP (n = 8), young FIP (n = 10-12), aged sham (n = 3), aged naïve (n = 4-6), young sham (n = 3), and young naïve (n = 4-6). Data are presented as mean ± SD from aged FIP and the data was analyzed using a mixed-effects analysis. P-values < 0.05 were considered significant.

As shown in Fig. 5(panels A–G), there were no differences in plasma levels of immunothrombosis biomarkers, lung MPO, and CCL2, between aged sham and naïve mice and young sham and naïve mice. Aged FIP non-survivors and young FIP survivors/non-survivors demonstrated elevated levels of IL-10 compared to age matched naïve and sham controls (Fig. 5B). Furthermore, aged FIP non-survivors demonstrated elevated levels of IL-6 compared to their age-matched naive controls (Fig. 5A). Aged FIP non-survivors also demonstrated elevated levels of CFDNA and CCL2 compared to their age-matched naïve and sham controls (Fig. 5E, F). Aged and young FIP non-survivors demonstrated decreased levels of ADAMTS13 activity compared to age-matched sham and naive controls (Fig. 5G). The single aged FIP survivor demonstrated an elevated level of IL-6 compared to young FIP survivors, suggesting persistent inflammation in the aged mice (Fig. 5A). Aged FIP non-survivors also demonstrated reduced levels of IL-10 and elevated levels of CFDNA compared to young FIP non-survivors (Fig. 5B and E). No difference in levels of lung MPO, TAT, CCL2, and ADAMTS13 activity was observed between aged and young FIP mice, in either survivors or non-survivors (Fig. 5C, D, F and G). Overall, these studies suggest that aged FIP mice exhibit a trend towards persistently elevated IL-6 (in survivors as well as non-survivors). In addition, aged FIP non-survivors exhibit reduced levels of IL-10 and elevated levels of CFDNA compared to young FIP non-survivors.

**Fig. 6:**
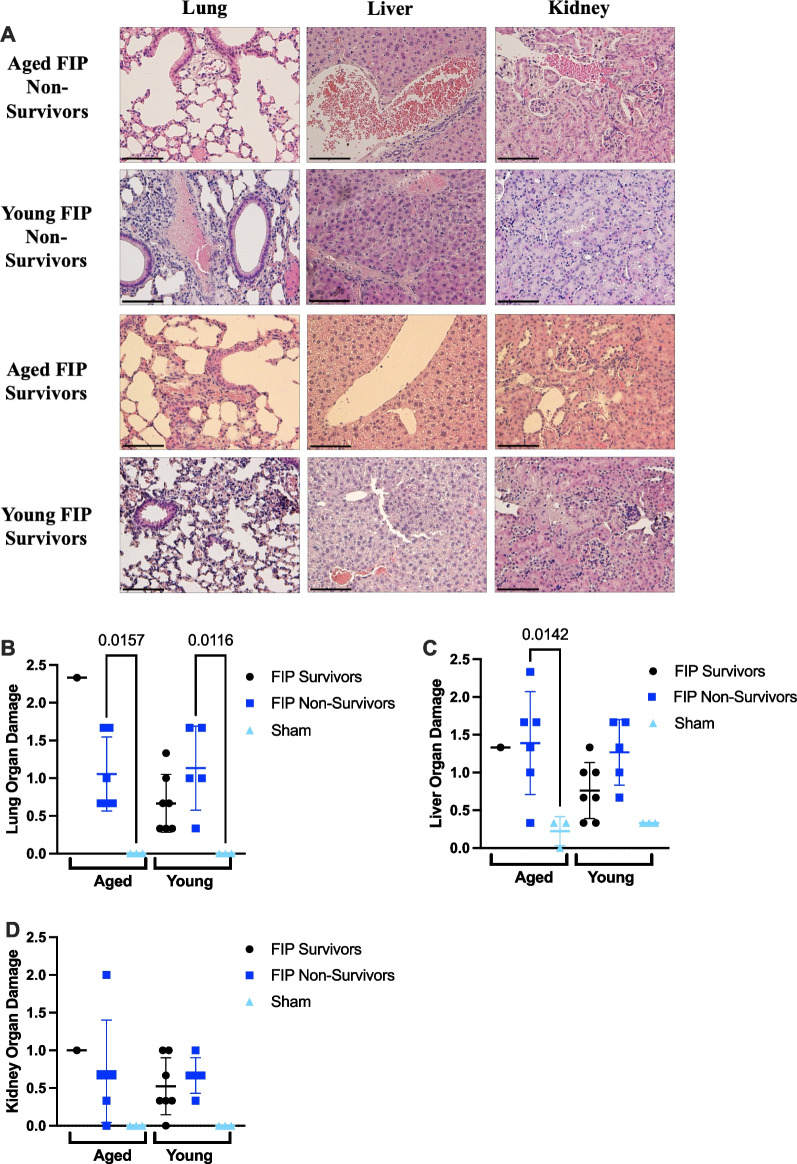
Histology scores in the lung, liver, and kidney in aged and young mice in a 72 h FIP model of sepsis. The lung, liver, and kidney of aged and young mice were harvested at humane or experimental endpoint, stained with H&E, and scored. Representative images of the lung, liver, and kidney for aged and young FIP mice are provided (**A**). Organ injury scores for the lung (**B**), liver (**C**), and kidney sections (**D**) were based on inflammation, thrombosis, and organ morphology. Mice that survived till experimental endpoint are designated as survivors. Mice that were humanely euthanized according to their MSS are designated as non-survivors. Aged FIP survivors (n = 1), aged FIP non-survivors (n = 6), aged sham mice (n = 3), young FIP survivors (n = 7), young FIP non-survivors (n = 5), and young sham mice (n = 3). Data are presented as mean ± SD and the data was analyzed using a one way-ANOVA. P-values < 0.05 were considered significant. Scale bars represent 50 $\mu$ m

Analysis of organ histology further revealed that no difference was observed in lung damage, liver damage, and kidney damage between aged FIP and young FIP mice (Fig. 6A–D).

## Discussion

Although age is an independent predictor of poor outcome in sepsis, very few preclinical animal studies use appropriately aged mice [6, 9]. In addition to differences in sepsis survival rates, age also influences the management of sepsis as well as the effectiveness of therapies. For example, the management of sepsis in the elderly requires considerations of factors such as resuscitation (ie. measures to improve cardiac output that focus on systolic function) and source control (ie. dosing of antimicrobials should factor in age-related differences in pharmacokinetic and pharmacodynamic parameters) [9]. Furthermore, the efficacy of anticoagulant therapies for septic patients with disseminated intravascular coagulation (DIC) may be associated with patients’ age [23]. Thus, incorporation of aged mice within the NPSP may help to identify age-related differences in sepsis pathophysiology as well as age-specific treatment strategies.

To our knowledge, this is the first study to investigate the impact of age on survival using a FIP model of abdominal sepsis. Using the standardized FIP model developed by the NPSP, we demonstrated that advanced age is associated with early organ injury and poor survival. The worsened organ injury and poor survival in aged mice may be attributed to heightened inflammation (IL-6) in aged mice in the early stage of sepsis (at 8 h) (Fig. 2A).

Our findings are consistent with previous studies demonstrating an age-related increase in mortality using CLP or endotoxemia models [8, 24–27]. Although our study was not powered to detect sex-differences in survival in the young and aged cohorts of mice, previous studies have shown an improved survival in females compared to males, which may reflect differences in hormonal and/or inflammatory responses [28]. Although the mechanisms are not fully understood, this sex-linked difference is also present in human sepsis, where female patients have lower mortality, shorter hospitalization, and ICU stays, and were less likely to require additional therapies such as dialysis or ventilation support [29, 30].

Monocyte chemoattract protein (MCP-1)/CCL2 is a chemokine that plays a role in recruiting monocytes, macrophages, and lymphocytes to help reduce bacterial burden [31]. In the 72 h survival study, we observed that aged FIP non-survivors exhibited a trend towards higher bacterial loads (Fig. 4E, F) and elevated levels of CCL2 (Fig. 5F) compared to young FIP non-survivors, a finding consistent with previous studies using CLP [8, 11, 31]. Age-related impairment in bacterial clearance may be attributed to reduced chemotaxis of myeloid cells, decreased phagocytosis, and an inability to upregulate certain genes related to innate immunity [8, 11, 31].

We further investigated the development of inflammation, coagulation, and organ injury in aged FIP mice, young FIP mice, and naïve/sham mice in our 72 h model of sepsis. Previous models of sepsis have shown elevated levels of inflammatory cytokines in aged septic mice compared to young septic mice [6]. We observed a trend towards elevated levels of IL-6 between aged FIP mice and young FIP mice, a finding consistent with previous in vivo studies using the CLP model (Fig. 5A) [6, 24, 32]. We also observed higher CFDNA levels in aged FIP non-survivors compared to young FIP non-survivors (Fig. 5E), which may contribute to the earlier time of death of aged FIP mice (Fig. 4A). Previous studies have shown that elevated levels of CFDNA in ICU patients with sepsis increases blood coagulation and is a strong predictor of poor outcome [33, 34]. Comprehensive studies of the methylation patterns of CFDNA have shown that plasma CFDNA in septic patients originates mainly from neutrophils as well as injured hepatocytes [35].

In this study, we also measured activity levels of ADAMTS13, a circulating metalloprotease that regulates the platelet-binding capacity of VWF [36]. We observed a trend towards decreased ADAMTS13 activity in the aged and young FIP mice compared to age-matched sham and naïve mice (Fig. 5G). This finding is consistent with our previous observational study of ADAMTS13 activity levels in ICU patients with sepsis [37]. Acquired deficiency of ADAMTS13 likely reflects the acute release of VWF in response to infection-induced inflammation, which consumes limited amounts of circulating ADAMTS13 [37, 38]. In humans, the VWF/ADAMTS13 ratio in bacteremia patients correlates with a more severe disease state [38]. In a mouse model of *S. aureus* sepsis, ADAMTS13 deficiency increased microvascular thrombosis and reduced survival, which could be rescued by the administration of recombinant ADAMTS13 [38].

Some of the strengths of this study include the use of both sexes of mice as well as the addition of clinically relevant therapies in our FIP model (ie. antibiotics and fluid resuscitation). We also used a diet-restriction model of aging to avoid the confounding effect of obesity with age. During aging, mice presented with unrestricted food tend to develop obesity [18, 39, 40]. Obesity has been shown to worsen the inflammatory response in septic mice [18, 39, 40]. Therefore, by diet-restricting our aged mice, we were able to examine the effects of sepsis on age, without the confounding effect of obesity. A limitation of the study is the use of a small number of aged mice (n = 8 for the time course study and n = 9 for the 72 h study), primarily due to the high cost involved in housing the diet-restricted mice for up to 12 months. As a result, the study was not adequately powered to detect differences for some of the clinical outcomes (such as whether biological sex impacts survival). Thus, future work examining these outcomes in a time course study and 72 h survival study with a larger sample size would be important to strengthen our findings.

In summary, using the standardized FIP model developed by the NPSP, we demonstrate that aged mice exhibit greater organ injury and a higher mortality rate compared to young mice. This difference may reflect increased inflammation in the early state of sepsis in aged mice. We also observed trends in impaired bacterial clearance, increased coagulation, and decreased ADAMTS13 activity in aged septic mice. Our aging model may help to increase the scientific validity of preclinical sepsis research and may be useful for identifying mechanisms of age-related susceptibility to sepsis as well as age-specific treatment strategies.

### Supplementary Information

**Additional file 1: Table S1.** Endpoint monitoring description form. **Table S2.** Outline of experimental studies. **Table S3.** Histology Scoring. **Figure S1.** Sex-related differences in MSS, temperature, and bacterial loads. in a 72h FIP model of sepsis.

**Additional file 2.** The ARRIVE guidelines 2.0: author checklist.

## Data Availability

Further data available upon reasonable request.

## Abbreviations

ADAMTS13: A disintegrin and metalloproteinase with a thrombospondin type 1 motif, member 13
CCAC: Canadian Council on Animal Care
CIHR: Canadian Institutes of Health Research
CLP: Cecal ligation and puncture
CFDNA: Cell-free DNA
DIC: Disseminated intravascular coagulation
FIP: Fecal-induced peritonitis
H&E: Hematoxylin and eosin
IL: Interleukin
IP: Intraperitoneal
LPS: Lipopolysaccharide
MSS: Murine sepsis score
(MCP-1)/CCL2: Monocyte chemoattract protein
MPO: Myeloperoxidase
NPSP: National Preclinical Sepsis Platform
NETs: Neutrophil extracellular traps
ANOVA: Analysis of variance
PBS: Phosphate buffered saline
PCF: Peritoneal cavity fluid
SD: Standard deviation
TAT complex: Thrombin-antithrombin complex
TaARI: Thrombosis and Atherosclerosis Research Institute
VWF: Von Willebrand factor
