# Supplementary material for: Impact of age on the host response to sepsis in a murine model of fecal-induced peritonitis
Source: Intensive Care Med Exp. 2024 Mar 8;12:28. doi: 10.1186/s40635-024-00609-8 (PMC10923763; doi:10.1186/s40635-024-00609-8)
Supplement: Supplementary file 1 — Additional file 1: Table S1. Endpoint monitoring description form. Table S2. Outline of experimental studies. Table S3. Histology Scoring. Figure S1. Sex-related differences in MSS, temperature, and bacterial loads. in a 72h FIP model of sepsis. [file 40635_2024_609_MOESM1_ESM.docx]

**Additional file 1**

|  | Score | | | |
| --- | --- | --- | --- | --- |
|  | **0** | **1** | **2** | **3** |
| Murine Sepsis Score (MSS) | | | | |
| Posture | Slim body | Hunched slightly | Hunched | Hunched huge |
| Activity | Normal  10 + steps | Reduced  <10 steps | Little (even when provoked)  1 – 2 steps | Stationary (even when provoked)  0 steps |
| Respiration Quality | Normal | Periods of labored (slow/fast) breathing | Consistently labored (slow/fast) breathing | Labored breathing with gaps  (slow/fast) breathing |
| Responsiveness | Normal; Fast response to auditory or touch stimuli | Slowed response to auditory or touch stimuli | No response to auditory, slowed response to touch | No response to touch stimuli |
| Ruffled Fur | Normal coat; Smooth fur | Slightly ruffled fur (25% of body) | Majority of fur is ruffled (50-75% of body) | Ruffled fur and piloerection  (100% of body) |

**Table S1: Endpoint Monitoring Description Form.**

A sample endpoint monitoring description form was used during our post-operative monitoring to assess sepsis progression via the murine sepsis score. Components are given a score from 0 to 3 (0 representing characteristics similar to those in healthy control mice and 3 being the most severe) [19, 25].

| **Experimental Studies** | **Biomarkers** | **Histology** | **Figures** |
| --- | --- | --- | --- |
| Cohort 1: Time Course Study (12h) | Conducted at:  4h, 8h, and 12h post-FIP/dextrose injection | Conducted at:  8h post-FIP/dextrose injection | Figure 1: MSS, body temperature, and bacterial loads  Figure 2: Biomarker data  Figure 3: Histology scores |
| Cohort 2: 72h Study | Conducted at:  72 h (Mice that survived till the experimental endpoint, (72h) were designated as survivors)  Before 72h (Mice that needed to be humanely euthanized before 72h were designated as non-survivors) | Conducted at:  72 h (Mice that survived till the experimental endpoint, (72h) were designated as survivors)  Before 72h (Mice that needed to be humanely euthanized before 72h were designated as non-survivors) | Figure 4: Mortality, MSS, body temperature, and bacterial loads  Figure 5: Biomarker data  Figure 6: Histology scores |

**Table S2: Outline of Experimental Studies.** An overview of the experimental studies, including the 2 cohorts of mice (12h time course study and 72h study), and time of biomarker and histology are provided.

**
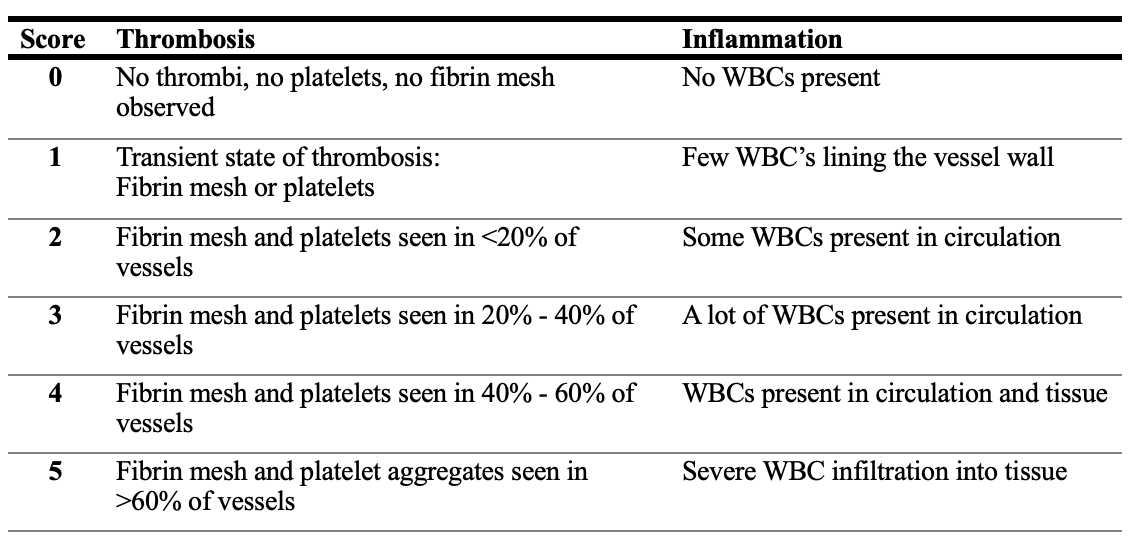
**

**Table S3: Histology Scoring.** A histology scoring sheet was used to score the liver, lung, and kidney. Components are given a score from 0 to 5 (0 representing characteristics similar to those in healthy control mice and 5 being the most severe) (Medeiros et al., 2023).

**Figure S1: Sex-related differences in MSS, temperature, and bacterial loads. in a 72h FIP model of sepsis.**

MSS over time for males (A) and females (B), body temperature over time for males (C) and females (D), and bacterial loads for aged and young survivors (mice that survived till the experimental endpoint (72h) are designated as survivors) and non-survivors (mice that were humanely euthanized during the 72h study according to their MSS are designated as non-survivors). Aged FIP (n = 9; male 5, female 4), Young FIP (n = 12; male 6, female 6), Aged Sham (n = 3; male 2, female 1), Young Sham (n = 3; male 2, female 1). Data are presented as mean ± SD. Bacterial loads for PCF (E) and blood (F) in aged and young FIP survivors and non-survivors. (Note: For 1 aged FIP non-survivor, unable to collect blood and for 1 young FIP survivor, blood and PCF colonies were too confluent to count).
